# The Early Activation Marker CD69 Regulates the Expression of Chemokines and CD4 T Cell Accumulation in Intestine

**DOI:** 10.1371/journal.pone.0065413

**Published:** 2013-06-12

**Authors:** Katarina Radulovic, Valerio Rossini, Calin Manta, Karlheinz Holzmann, Hans A. Kestler, Jan Hendrik Niess

**Affiliations:** 1 Department of Internal Medicine I, University of Ulm, Ulm, Germany; 2 Institute for Microbiology and Biotechnology, University of Ulm, Ulm, Germany; 3 Microarray Core Facility, University of Ulm, Ulm, Germany; 4 Research Group Bioinformatics and Systems Biology, Institute of Neural Information Processing, University of Ulm, Ulm, Germany; 5 Department of Visceral Surgery and Medicine, Inselspital, Bern, Switzerland; Charité-Universitätsmedizin Berlin, Germany

## Abstract

Migration of naïve and activated lymphocytes is regulated by the expression of various molecules such as chemokine receptors and ligands. CD69, the early activation marker of C-type lectin domain family, is also shown to regulate the lymphocyte migration by affecting their egress from the thymus and secondary lymphoid organs. Here, we aimed to investigate the role of CD69 in accumulation of CD4 T cells in intestine using murine models of inflammatory bowel disease. We found that genetic deletion of CD69 in mice increases the expression of the chemokines *CCL-1*, *CXCL-10* and *CCL-19* in CD4^+^ T cells and/or CD4^−^ cells. Efficient *in vitro* migration of CD69-deficient CD4 T cells toward the chemokine stimuli was the result of increased expression and/or affinity of chemokine receptors. *In vivo* CD69^−/−^ CD4 T cells accumulate in the intestine in higher numbers than B6 CD4 T cells as observed in competitive homing assay, dextran sodium sulphate (DSS)-induced colitis and antigen-specific transfer colitis. In DSS colitis CD69^−/−^ CD4 T cell accumulation in colonic lamina propria (cLP) was associated with increased expression of *CCL-1*, *CXCL-10* and *CCL-19* genes. Furthermore, treatment of DSS-administrated CD69^−/−^ mice with the mixture of CCL-1, CXCL-10 and CCL-19 neutralizing Abs significantly decreased the histopathological signs of colitis. Transfer of OT-II×CD69^−/−^ CD45RB^high^ CD4 T cells into RAG^−/−^ hosts induced CD4 T cell accumulation in cLP. This study showed CD69 as negative regulator of inflammatory responses in intestine as it decreases the expression of chemotactic receptors and ligands and reduces the accumulation of CD4 T cells in cLP during colitis.

## Introduction

In the intestinal immune system chemokine ligands and receptors regulate the migration of lymphocytes. Naïve cells express high levels of L-selectin (CD62L) and chemokine receptor CCR-7 that recognize secondary lymphoid organs (SLO)-expressed addressin and the chemokines CCL-19 and CCL-21, respectively [1], [2], [3]. Lymphocyte egress from the SLO depends on the expression of sphingosine 1-phosphate receptor type 1 (S1P_1_) on the lymphocyte surface and its interaction with the ligand sphingosine 1-phosphate (S1P) that is abundant in the lymph [4], [5]. Activated lymphocytes express different combinations of chemokine receptors depending on their migration destination and the subtype they differentiate to. For example Th1 cells express CXCR-3 (binding CXCL-10) [6], Th17 cells express CCR-6 (binding CCL-20) [7] while CCR-8 (binding CCL-1) is implicated in Th2 responses but studies showed its expression primarily on the memory cells of Th2 subtype and Foxp3 regulatory (Treg) cells [8]. Lymphocytes that migrate from the SLO to the gut express the chemokine receptor CCR-9 and the integrin α4β7 that bind CCL-25 and mucosal addressin cell adhesion mlecule-1 (MadCAM-1), respectively [1], [2].

Inflammatory bowel disease (IBD), such as Crohn’s disease (CD) and ulcerative colitis (UC) are thought to be the product of a deregulated immune response to constituents of the intestinal microflora. The migration of lymphocytes to the lamina propria seems to be a key event for the pathogenesis of IBD. Strategies that block the recruitment of leukocytes into the intestine represent a potentially potent treatment of IBD. The anti-α4 mAb was efficient in the treatment of CD [9] but it increases the susceptibility to any infection showing the need for tissue specific migration inhibitor. FTY-720 (fingolimid) as the agonist of S1P receptor family was very effective in the animal models of IBD [10], [11], but it showed the side effect of bradycardia in the clinical trials [12] due to expression of S1P_3_ receptor on myocard [13]. Studies are now being conducted with the agonists specific only for S1P_1_ receptor expressed exclusively on lymphocytes [13] and some of them are promising in the IBD treatment [14], [15]. Also the mAb to CXCL-10 as the agent that specifically inhibit the migration of Th1 cell subset, is in the clinical trials for the treatment of UC [9].

The C-type lectin receptor CD69 (encoded in NK gene cluster) is the earliest activation antigen of lymphocytes. This molecule is shown to be involved in the regulation of immune responses in murine models of asthma [16], [17], arthritis [18], [19], colitis [20], myocarditis [21], pathogen clearance [22] and tumors [23], [24]. CD69 activation induces TGF-β expression and suppresses the production of pro-inflammatory cytokines IL-17 and IFN-γ [19], [20], [23], [25], [26]. Studies showed that activation of CD69 leads to ERK phosphorylation and thus stabilizes TGF-β on the cell surface of lymphocytes [27]. After allogenic bone-marrow transplantation CD4**^+^**CD69^+^CD25^−^ T cells protect from the development of graft-versus host disease [28]. CD69^+^ T cells are able to induce indoleamine 2,3-dioxygenase (IDO) in tumor-associated macrophages and hence down-regulate inflammatory immune responses [29]. Therefore CD4**^+^**CD69^+^CD25^−^ T cells have been introduced as novel regulatory cell type whose effector functions depend mainly on TGF-β.

Besides regulating the cytokine response, CD69 has also been shown to affect the migration of immune cells. Constitutive overexpression of CD69 in transgenic animals implicated a role of this molecule in releases of lymphocytes from the thymus [30], [31]. Furthermore, CD69 directly interact with S1P_1_ receptor on the lymphocyte surface inducing the down-regulation of S1P_1_ expression [32], [33]. Consequently, CD69 mediates the retention of activated lymphocytes in the SLO [4], [32]. The migration of dendritic cells (DC) from the skin to draining lymph nodes is increased in CD69-deficient animals [34]. Overall, these studies suggested an important role of CD69 in regulating the migration of lymphocytes, but the molecular mechanism of this effect is not completely solved.

This study showed that CD69-deficiency increases *CCL-1*, *CXCL-10* and *CCL-19* expression. CD69-defiecient CD4 T cells migrated rapidly toward the chemokine gradient *in vitro* and accumulated in the intestinal tissues *in vivo*. Furthermore, CD69^−/−^ animals showed increased fraction of naïve and decreased fraction of memory CD4 T cells compared to B6 animals. Absence of CD69 induced severe intestinal inflammation in DSS-induced colitis and antigen-specific transfer colitis. Severity of the colitis could be ameliorated in DSS-administrated CD69-deficeint mice with the neutralization of CCL-1, CXCL-10 and CCL-19. These data indicated that CD69 is an important regulator of immune responses that regulates the expression of chemokines and cell accumulation during intestinal inflammation.

## Materials and Methods

### Mice

Inbred C57BL/6J (B6) mice, RAG^−/−^ (RAG^tm1Mom^), CD69^−/−^ mice [17], [18], [20], B6.Cg-Tg (CAG-DsRed*MST) 1 Nagy/J (in text referred as DsRed mice) [35], T cell receptor (TCR)-transgenic OT-II, OT-II×CD69^−/−^ [20] and OT-II×DsRed all on CD45.2 background, as well as B6 mice on CD45.1 background (B6.SJL-*Ptprc^a^ Pep3^b^*/BoyJ ) [36] were bred and kept under specific pathogen-free (SPF) conditions in the animal facility of Ulm University (Ulm, Germany). Female and male mice were used at 6–12 weeks of age. The local animal committee (Regierungspräsidium Tübingen) approved the animal protocols (protocol no. #1010, #1067 and #1089). All animal experiments were performed according to the guidelines of the local Animal Use and Care Committee and the National Animal Welfare Law.

### Dextran Sodium Sulphate (DSS)-induced Colitis

B6 and CD69^−/−^ mice were fed 2% DSS salt (cat. no. 160110; MP Biomedicals, Illkirch, France) dissolved in sterile water ad libitum for five days. On day 5, DSS containing water was removed and replaced with normal sterile water. In one experiment mice were treated with chemokine antagonists (anti-CCL-1 Ab 148113 (cat. no. MAB845), anti-CXCL-10 Ab 134013 (cat. no. MAB466) and anti-CCL-19 Ab 87201 (cat no. MAB880) all purchased from R&D Systems, Wiesbaden, Germany). The mixture of the respective anti-chemokine Abs was injected i.p. on days 0, 2, 4 and 6 after DSS administration with the concentration of the individual Ab being 1 mg/kg. The weight of mice and their clinical condition were monitored daily. Mice were sacrificed at day 3 or at the peak of disease, at day 7 after DSS administration. Tissue samples for histopathological examination were taken from the large intestine, fixed in neutral-buffered formalin, embedded in paraffin, sectioned on a microtome, mounted on slides, and stained with hematoxylin and eosin (H&E). Histology of the large intestine was categorized as normal (score 0); mild colitis (score 1), with few inflammatory cells in the lamina propria (LP), stromaedema, and a slight reduction of goblet cells; moderate colitis (score 2), with an intense inflammatory infiltration of the LP, hyperplasia of crypts, and a marked reduction of goblet cells; or severe colitis (score 3), with a spillover of leukocytes beyond the mucosa into deeper layers of the colonic wall, complete loss of goblet cells, distortion of the mucosal architecture, erosions or ulcerations, and crypt abscesses as previously published [20], [37], [38].

### Antigen-specific Transfer Colitis Model

Total spleen cells of OT-II or OT-II×CD69^−/−^ mice were isolated and stained for CD4 and CD45RB with FITC- conjugated mAb binding CD4 GK1.5 (cat. no. 11-0041-86; eBioscience, Frankfurt, Germany) and biotinylated mAb binding CD45RB 16A (cat. no 553093; BD Biosciences, Heidelberg, Germany), followed by the second-step reagent Streptavidin-Peridinin Chlorophyll Protein (PerCP)-Cy5.5 (cat. no. 45-4317-80; eBioscience). CD45RB^high^ T cells from the CD4^+^ population were enriched using the FACSAria system (BD Biosciences) to a purity >95%. Purified CD45RB^high^ CD4 T cells were injected i.p. into RAG^−/−^ mice (3×10^5^ cells/mouse). Hosts were fed or not intragastrically every second day with 1 mg of chicken ovalbumin (OVA) protein (cat. no. A5253; grade II, Sigma-Aldrich, Steinheim, Germany) dissolved in 100 µl of PBS. The weight of transplanted mice and their clinical condition were monitored twice weekly. Tissue samples for histopathological examination were taken from the large intestine, fixed in neutral-buffered formalin, embedded in paraffin, sectioned on a microtome, mounted on slides, and stained with H&E. Histological scoring was preformed same as described above for the DSS colitis model.

### *In vitro* Chemokine Receptor Functionality Assay

Chemokine stocks were made in PBS supplemented with 0.1% of BSA and diluted in RPMI medium containing 10% FBS and 1% Penicillin/Streptomycin when used for experiments. Concentrations of CCL-1 (cat. no. 845-TC; R&D Systems), CXCL-10 (cat. no. 466-CR; R&D Systems), CCL-5 (cat. no. 478-MR; R&D Systems) and CCL-4 (cat. no. 451-MB; R&D Systems) were titrated (from 0.5 nM to 200 nM) and 600 µl of only medium or medium containing chemokine was added in the lower chamber of 24-well transwell system with 5 µm pore size polycarbonate membrane (cat. no. 3421; Corning, New York). This membrane does not allow the medium to diffuse, but allows T cells to migrate from one chamber to another. After loading the lower chamber, inserts containing the membrane were carefully placed into the wells. CD4 T cells were enriched from the total spleen cells of B6 and CD69^−/−^ mice using the MACS CD4 T cell isolation kit (cat. no. 130-090-860; Miltenyi Biotec, Bergisch Gladbach, Germany). 4×10^5^ B6 or CD69^−/−^ CD4 T cells in 100 µl of RPMI, 10% FBS, 1% Penicillin/Streptomycin medium were added to the upper chambers. Plate was covered and incubated 1 hour at 37°C, 5% CO_2_. Transwell inserts were carefully removed and cells in the lower chamber media counted by FACSCalibur. All reactions were done in at least four repeats. The chemotactic index was calculated as: the number of cells migrating to the media with chemokine divided by the average number of cells migrating to the media alone as previously described [39].

### *In vivo* Competitive Homing Assay

CD4 T cells were enriched via MACS CD4 T cell isolation kit from the spleens of DsRed or CD69^−/−^ mice (both on B6 CD45.2^+^ background). CD69^−/−^ CD4 T cells were labelled with cell trace dye carboxyfluorescin succinimidyl ester (CFSE, cat. no. C34554; Invitrogen, Darmstadt, Germany): 10^7^ cells were washed two times with PBS, resuspended in 1 ml of PBS, 0.1% BSA and than incubated 15 min on 37°C, 5% CO_2_ with 5 µM final concentration of CFSE diluted in DMSO; labelling reaction was stopped by adding 1 ml of cold FBS and washing the cells two times with PBS. DsRed B6 and CSFE^+^ CD69^−/−^ CD4 T cells were mixed in a 1∶1 ratio and resuspended in PBS. 10^6^ of each cell population in 200 µl of PBS were i.v. transferred to CD45.1^+^ B6 mice. Small amount of cells was saved after injection for calculating input ratio (IR). These cells were extracellulary stained for CD4 with APC-conjugated mAb binding CD4 GK1.5 (cat. no. 17-0041-83; eBioscience) and for CD45.2 with biotinylated anti-CD45.2 mAb 104 (cat. no. 553771; BD Bioscience) and PerCP-Cy5.5 as a second-step reagent. Cells were analyzed by Flow Cytometry (FCM) and IR was calculated as:

IR = number of CD4^+^CD45.2^+^CFSE^+^ cells/number of CD4^+^CD45.2^+^DsRed^+^ cells.

Transferred hosts were euthanized 18 or 72 h after injection. Cells were isolated from the mesenteric lymph nodes (MLN), blood, small intestinal lamina propria (siLP) and colonic lamina propria (cLP) of the hosts and stained same as the sample before injection (with APC-conjugated mAb binding CD4 GK1.5, biotinylated mAb binding CD45.2 104 and PerCP-Cy5.5 as a second-step reagent). Samples were analyzed by FCM and homing index (HI) for every tissue calculated as:

HI = number of CD4^+^CD45.2^+^CFSE^+^ cells/number of CD4^+^CD45.2^+^DsRed^+^ cells : IR.

As suggested [39] this index was normalized to the HI in the blood to eliminate possible retention of the one cell type in some of the secondary lymphoid tissues which could eliminate that cell type from the circulation.

In a separate experiment 1×10^7^ of each cell population in 200 µl PBS were i.v. injected to one B6 mouse. 18 h post-injection small intestine and colon were isolated and washed from faeces with PBS/1% FBS using tubing needle. Washed small intestine and colon were incubated for 1 h at room temperature in dark with Alexa Fluor 350-conjugated Wheat Germ Agglutinin (WGA, cat. no. W11263; Invitrogen), 20 µg/ml final concentration, in 1 ml PBS/1%FBS. WGA binds mucus glycoproteins in the intestine [40] staining for the surface of intestinal epithelium. Small intestinal and colonic tissues were analyzed by LSM 710 Confocal Laser Scanning Microscope (Zeis, Jena, Germany) and different fluorescent colours were detected using following excitation/emission spectra: for Alexa Fluor 350 350 nm/408–464 nm, for DsRed 512 nm/591–715 nm and for CFSE 488 nm/493–525 nm. Pictures were obtained at the original magnification 40x/1.30 oil DIC M27 and were analyzed using LSM Image Browser software (Zeis, Jena, Germany).

### Antigen-specific *in vivo* Competitive Homing Assay

CD4 T cells from the spleen of OT-II×DsRed or OT-II×CD69^−/−^ mice (both on Vβ5^+^ background) were enriched by MACS and OT-II×CD69^−/−^ CD4 T cells were labelled with CFSE by the protocol described in previous section. OT-II×DsRed and CSFE^+^ OT-II×CD69^−/−^ CD4 T cells were mixed in a 1∶1 ratio and 10^6^ of each cell population in 200 µl of PBS were i.v. transferred to B6 hosts. A small amount of cells was saved after injection for calculating IR. These cells were extracellulary stained for CD4 with APC-conjugated mAb binding CD4 GK1.5 and for Vβ5 with biotinylated anti- Vβ5.1, 5.2 mAb MR9-4 (cat. no. 553188; BD Bioscience) and PerCP-Cy5.5 as a second-step reagent. Cells were analyzed by FCM and IR was calculated as:

IR = number of CD4^+^ Vβ5^+^CFSE^+^ cells/number of CD4^+^ Vβ5^+^DsRed^+^ cells.

Hosts were fed or not intragastrically with 1 mg OVA protein in 100 µl PBS daily. 72 h after injection hosts were euthanized and cells from the MLN, blood, siLP and cLP were isolated and stained same as the IR sample (with APC-conjugated mAb binding CD4 GK1.5, biotinylated mAb binding Vβ5.1, 5.2 MR9-4 and PerCP-Cy5.5 as a second-step reagent). Cells were analyzed by FCM and HI for every tissue calculated as:

HI = number of CD4^+^ Vβ5^+^CFSE^+^ cells/number of CD4^+^ Vβ5^+^DsRed^+^ cells : IR.

As before, this HI was normalized to the HI of the blood.

### Microarray Data

Gene expression data of B6, CD69-activated B6 and CD69^−/−^ CD4 T cells have analyzed as previously described by our group [20]. These data are accessible at NCBI GEO database (GEO; accession number GSE27706).

### CD4 T cell Isolation

CD4 T cells were isolated from the blood, cLP, siLP, spleen and MLN of B6, CD69^−/−^, DsRed, OT-II, OT-II×CD69^−/−^, OT-II×DsRed and CD45.1 B6 mice.

#### Isolation of blood cells

Arterial blood was obtained from the mice, mixed with EDTA as anti-coagulant and red blood cells were lysed by 3 times 5 min incubation on 37°C with 5 ml of Lysebuffer (0.16 M NH_4_Cl and 0.17 M Tris, pH 7.2) followed by washing with PBS/1%FBS.

#### Isolation of spleen and MLN cells

Single-cell suspensions were aseptically prepared from spleen and MLN and red blood cells from spleen were lysed by 10 min incubation on 37°C with 5 ml of Lysebuffer. All the cells were washed and resuspended in PBS supplemented with 1% FBS.

#### Isolation of small intestinal and colonic lamina propria cells

Segments of the small intestine or colon were washed with PBS to remove debris and mucous. The epithelium was removed by incubation at 37°C for 10–15 min under gentle shaking with 1 mM dithiothreitol (and 1 mM EDTA for colon tissue) in 25 ml PBS supplemented with 1% FBS. The remaining tissue was washed in PBS to remove residual epithelial cells, and the supernatants were discarded. Intestinal tissues were cut into 2×2-mm pieces and digested by incubation with 0.25 mg/ml collagenase type VIII from Clostridium histolyticum (cat. no. C-2139; Sigma-Aldrich, St. Louis, MO) for 30–45 min at 37°C in RPMI 1640 under shaking. Supernatants were collected, from which LP lymphocytes were pelleted. LP lymphocytes were resuspended in RPMI 1640 medium containing 35% Percoll (density 1.124 g/ml; cat. no. L-6145; Biochrome, Berlin, Germany). This cell suspension was overlaid onto 70% Percoll and centrifuged for 20 min at 750×g. Viable cells at the 35%/70% Percoll interface were collected and washed twice.

### Extracellular Staining

Cells were washed twice in PBS supplemented with 0.3% BSA and 0.1% sodium azide. Non-specific binding of antibodies to Fc receptors was blocked by preincubation of cells with mAb 2.4G2 (cat. no. 01241D; BD Biosciences) directed against the FcγRIII/II CD16/CD32 (0.5 ng mAb/10^6^ cells). Cells were washed and incubated with 1 ng/10^6^ cells of the relevant mAb for 20 min at 4°C and washed again twice. In some experiments, cells were subsequently incubated with 1 ng/10^6^ cells of the second-step reagent for 20 min at 4°C. The following antibodies were used: APC-conjugated mAb binding CD4 GK1.5 (cat. no. 17-0041-83; eBioscience, Frankfurt, Germany), CXCR3 CXCR3-173 (cat. no. 17-1831-80; eBioscience) and CCR7 4B12 (cat. no. FAB3477A; R&D systems); FITC-conjugated mAb binding CD4 GK1.5 (cat. no. 11-0041-86; eBioscience) and CD44 IM7 (cat. no. 553133; BD Biosciences); PE-conjugated mAb binding CD103 M290 (cat. no. 557495; BD Bioscience) and α4β7 DATK32 (cat. no. 553811; BD Bioscience); biotin-conjugated mAb binding CD62L MEL-14 (cat.no. 13-062-85; eBioscience) and CCR9 eBioCW1.2 (cat. no. 13-1991-81; eBioscience). As a second-step reagent PerCP-Cy5.5 was used.

### Intracellular Foxp3 Staining

Cells were isolated, washed with PBS supplemented with 0.3% BSA and 0.1% sodium azide and stained extracellularly with FITC-conjugated anti-CD4 mAb binding CD4 GK1.5 (cat. no. 11-0041-86; eBioscience). Surface-stained cells were than pelleted and resuspended in 200 µl of Fixation/Permeabilization buffer (cat. no. 00-5123-43; eBioscience). After overnight incubation at 4°C in the dark, cells were washed two times with 1×Permeabilization buffer (cat. no. 00-8333-56; eBioscience) and incubated for 15 min, 4°C with mAb 2.4G2 directed against the FcγRIII/II CD16/CD32 (0.5 ng mAb/10^6^ cells) for preventing non-specific binding of the Abs. Cell were pelleted and after discarding the supernatant incubated for 30 min at 4°C, dark with 0.5 ng/10^6^ cells of the PE-conjugated anti–Foxp3 FJK-16s (cat. no. 12-5773-80; eBioscience) mAb diluted in 1×Permeabilization buffer. Stained cells were washed once with 1×Permeabilization buffer, once with PBS supplemented with 0.3% BSA and 0.1% sodium azide, resuspended in PBS supplemented with 0.3% BSA and 0.1% sodium azide and analyzed on FCM.

### Cytokine Detection by ELISA

The concentration of IL-17 in the blood serums was measured by a conventional double-sandwich ELISA. Capture mAb TC11-18H10 (cat. no. 555068) and detection biotinylated mAb TC11-8H4.1 (cat. no. 555067) were purchased from BD Bioscience. Extinction was measured at 450 nm on a TECAN microplate-ELISA reader using EasyWin software (both from Tecan, Wetzlar, Germany).

### Chemokine and Cytokine Expression Analyses by qRT-PCR

RNA was prepared from approximately 30 mg of frozen tissue samples (MLN, small intestinal or colon tissue) or from 10^7^ fresh spleen cells (sorted by MACS for CD4^+^ and CD4^−^ fraction) using the RNAeasy mini kit (cat. no. 74904; Qiagen, Hilden, Germany). Contaminating genomic DNA was eliminated from samples by treatment with RNAse – free DNAse I (cat. no. 1010395; Qiagen). A total of 2 µg of RNA isolated from tissues or 200 ng of RNA isolated from cells was reverse transcribed with SuperScript II Reverse Transcriptase (cat. no. 18064-014; Invitrogen) using random primers (cat. no. 48190-011; Invitrogen) according to the manufacturer instructions. SYBR Green qPCR Master mix (cat. no. PA-012-12; SABiosciences, Qiagen) was used for amplification and detection. Real-time PCR reactions were performed using the 7500 Fast Real-Time PCR System (Applied Biosystems, Darmstadt, Germany) and the following conditions: 50°C 2 min, repeat 1; 95°C 10 min, repeat 1; 95°C 15 sec, 60°C 1 min, repeats 40; 95°C 15 sec, 60°C 1 min, 95°C 15 sec, 60°C 15 sec, repeat 1. *β-actin* PCR signals were used to equalize cDNA amounts between preparations. Following primers from SABioscience, Qiagen group were used: *β-actin* (cat. no. PPM02945A), *IFN-γ* (cat. no. PPM03121A), *CXCL-10* (cat. no. PPM02978D), *CCL-1* (cat. no. PPM03138B), *CCL-19* (cat. no. PPM03157B), *CCL-5* (cat. no. PPM02960F) and *CCL-8* (cat. no. PPM03165A). Expected products length was: for *β-actin* 154 bp, for *IFN-γ* 95 bp, *CXCL-10* 106 bp, *CCL-1* 92 bp, *CCL-19* 100 bp, *CCL-5* 96 bp and *CCL-8* 168 bp.

### Statistics

We performed Mann-Whitney test for assessing the difference of two samples and one-sample t-tests for testing the deviation from the theoretical mean (GraphPad Prism V4). The difference between data sets of three and more groups was analyzed with Kruskal-Wallis test and Mann-Whitney as post-hoc test (GraphPad Prism V4). P≤0.05 was considered statistically significant.

## Results

### Increased Expression of Chemokines in the Absence of CD69

To identify transcripts differentially regulated by the CD69 activation marker in CD4 T lymphocytes we carried out microarray analysis. We compared gene expression profiles of 3 groups: anti-CD3/CD28 activated B6 CD4 T cells; anti-CD3/CD28 activated CD69^−/−^ CD4 T cells and anti-CD3/CD28 activated B6 CD4 T cells on which surface CD69 molecule was activated by cross-linking. CD69 regulated the expression of chemokines as we observed the significant differences between our groups. Activation of CD69 significantly reduced the expression of *XCL-1*, *CCL-4*, *CCL-1*, *CCL-9*, *CCL-3*, *CXCL-9* and *CXCL-10* chemokine and *CCR-8*, *CCR-4*, *CCR-5* chemokine receptor transcripts in B6 CD4 T cells as compared to CD69^−/−^ CD4 T cells (**Table 1**). The expression of most chemokines was reduced in CD69-activated B6 CD4 T cells when compared to B6 CD4 T cells, but increased in CD69^−/−^ as compared to B6 CD4 T cells (**Table S1 and Table S2**).

**Table 1:** Expression of selected chemokine-related genes differentially expressed in CD69-activated B6 compared to CD69^−/−^ CD4 T cells analyzed by microarray.

Gene symbol	Description	Fold-change (log2)	FDR
Xcl1	chemokine (C motif) ligand 1	−3.09	5.48e-14
Ccl4	chemokine (C-C motif) ligand 4	−1.45	1.08e-05
Ccl1	chemokine (C-C motif) ligand 1	−1.00	1.49e-05
Ccl9	chemokine (C-C motif) ligand 9	−1.09	0.00016
Ccl3	chemokine (C-C motif) ligand 3	−1.45	5.48e-14
Cxcl9	chemokine (C-X-C motif) ligand 9	−1.56	5.48e-14
Cxcl10	chemokine (C-X-C motif) ligand 10	−1.75	5.48e-14
Ccr8	chemokine (C-C motif) receptor 8	−1.48	5.48e-14
Ccr4	chemokine (C-C motif) receptor 4	−1.07	4.48e-11
Ccr5	chemokine (C-C motif) receptor 5	−1.08	1.8e-06

Genes with a fold change (log2)≥1.00 and false discovery rate (FDR)≤0.05 were considered as differentially expressed.

**Figure 1:**
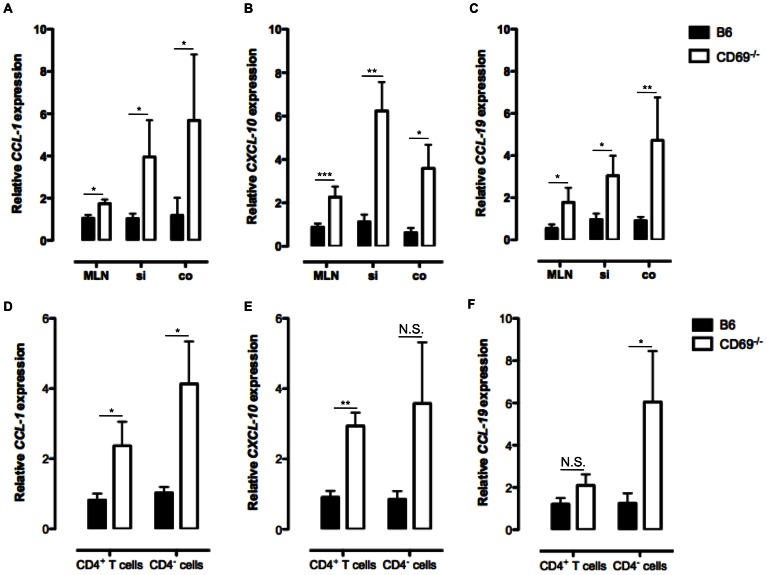
Absence of CD69 leads to the increased expression of *CCL-1*, *CXCL-10* and *CCL-19*. RNA was isolated from the frozen mesenteric lymph nodes (MLN), small intestinal (si) and colonic (co) tissue samples or from sorted CD4^+^ and CD4^−^ spleen cells of non-treated B6 or CD69^−/−^ animals and reverse transcribed to complementary DNA. Relative expression of *CCL-1* (**A** and **D**), *CXCL-10* (**B** and **E**) and *CCL-19* (**C** and **F**) genes compared to *β-actin* gene is analyzed by qRT-PCR. Mean (± SEM) for at least six mice per each strain is presented. *p≤0.05; **p≤0.005; ***p≤0.0005; N.S. – not significant.

To analyse if CD69 affects the expression of chemokines *in vivo*, we conducted qRT-PCR analyses. In absence of CD69 an increased expression of *CCL-1*, *CXCL-10* and *CCL-19* genes was observed in mesenteric lymph nodes (MLN), small intestinal (si) and colonic (co) tissues (**Fig. 1A–C**). To identify the cell source of increased chemokine production, RNA was isolated from CD4^+^ and CD4^−^ cells of B6 and CD69^−/−^ mice, reverse transcribed to cDNA and analysed by qRT-PCR. Confirming the micro-array data, increased expression of *CCL-1* and *CXCL-10* was found in CD69^−/−^ CD4^+^ T cells (**Fig. 1D,E**). CD69^−/−^ CD4^−^ cells showed increased expression of *CCL-1* and *CCL-19* but not of *CXCL-10* gene (**Fig. 1D–F**). However, no significant difference in expression of *CCL-5* and *CCL-8* was observed (**Fig. S1**). These data indicated that CD69 negatively regulates the expression of chemokines which protein products are involved in the migration of naive, activated and memory lymphocytes, in both CD4^+^ and CD4^−^ cell populations.

### CD69 Regulates the Migration of CD4 T cells Toward the Chemokine Gradient *in vitro*

**Figure 2:**
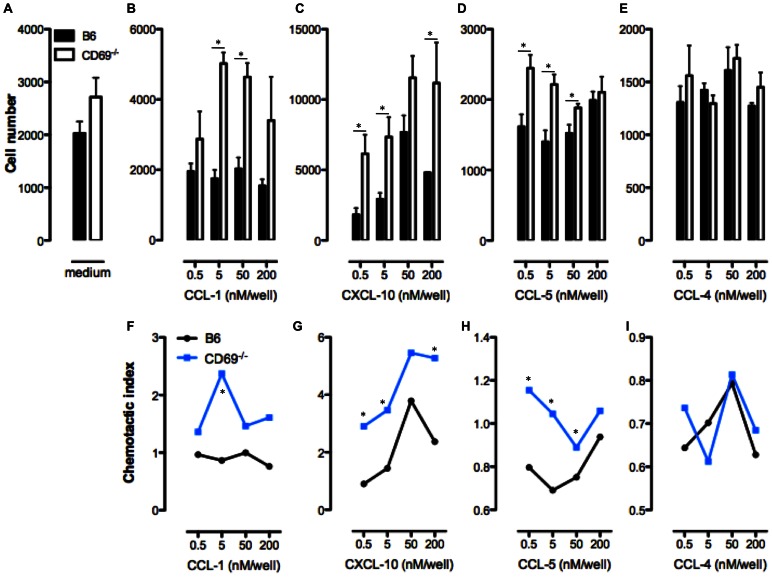
Increased *in vitro* functionality of CD4 T cell chemokine receptors for CCL-1, CXCL-10 and CCL-5 in the absence of CD69. CD4 T cells were enriched from the spleen of B6 or CD69^−/−^ mice and migration of these cells was analysed *in vitro* in transwell system. Medium alone or containing indicated concentrations of CCL-1, CXCL-10, CCL-5 and CCL-4 were loaded in the lower chamber of the transwell system, while CD4 T cells in the medium were loaded in the upper chamber. Cell number migrating from the upper chamber to the chemokine containing chamber trough polycarbonate membrane after 1 h at 37°C, 5% CO_2_ was counted by flow cytometry. All the reactions were done in at least four repeats and average cell number per chemokine concentration per cell type was calculated. Results are presented as the number of cells migrating to the wells containing medium alone (**A**) or indicated concentration of CCL-1 (**B**), CXCL-10 (**C**), CCL-5 (**D**) or CCL-4 (**E**). Mean (± SEM) for at least four repeats per each chemokine concentration and cell type is presented. The lower graph set represents chemotactic indexes of B6 and CD69^−/−^ CD4 T cells for CCL-1 (**F)**, CXCL-10 (**G)**, CCL-5 (**H**) and CCL-4 (**I**). The chemotactic index was calculated as: the number of cells migrating to the media with chemokine divided by the average number of cells migrating to the media alone. *p≤0.05.

As CD69 regulated the expression of chemokines, we investigated if CD69-deficiency affects the response of CD4 T cells toward the chemokine gradient *in vitro*. In this assay we used the transwell system with the polycarbonate membrane that prevents the diffusion of medium, but allows the migration of CD4 T cells in between the chambers. The lower chamber contained pure medium or the medium with titrated concentrations of chemokine. Upper chamber was filled with medium containing B6 or CD69^−/−^ spleen CD4 T cells. After 1 h incubation at 37°C, 5% CO_2_ the number of cells that migrated to the lower chamber was counted by FCM. There was no difference in the number of B6 and CD69^−/−^ CD4 T cells migrating to the medium alone (**Fig. 2A**). When CCL-1, CXCL-10 or CCL-5 were added to the medium significantly increased numbers of CD69-deficient as compared to B6 CD4 T cells were migrating toward the chemokine gradient for most of the chemokine concentrations tested (**Fig. 2B–D**). Chemotactic index represents the number of cells counted in the medium containing the chemokine normalized to the mean cell number counted in the pure medium and it was calculated for every chemokine concentration used. CD69^−/−^ CD4 T cells showed significantly increased chemotactic index for CCL-1, CXCL-10 and CCL-5 (**Fig. 2E–G**). Increased migration of CD69-deficient CD4 T cells toward CCL-1 and CCL-5 correlated with increased expression of the respective chemokine receptors for these chemokines, CCR-8 and CCR4/CCR5, respectively (**Table S2**). Significant differences between B6 and CD69^−/−^ CD4 T cells were not observed when the chemokine CCL-4 (another CCR4/CCR5 ligand) was used in our experiments (**Fig. 2E,I**). The expression of the CXCL-10 receptor, CXCR-3, by CD69^−/−^ CD4 T cells was not increased (**Fig. S2**). Thus, CD69-deficiency increases the chemokine-dependent migratory potential of CD4 T cells toward some, but not all chemokines.

### CD69 Regulates the Homing of CD4 T cells to the Intestinal Tissues *in vivo*

**Figure 3:**
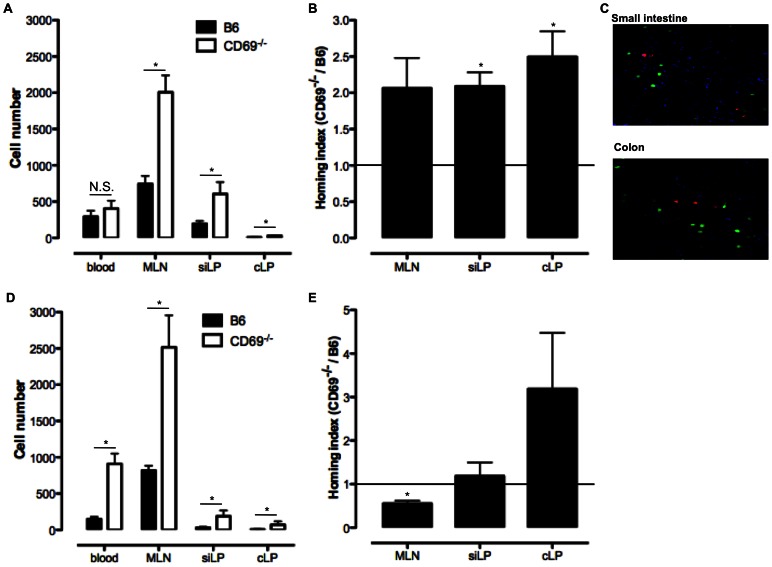
Increased migratory potential of CD69-deficient CD4 T cells to the mucosal intestinal tissues *in vivo*. CD4 T cells were enriched from the spleen of red fluorescent DsRed or CD69^−/−^ animals, both on the CD45.2^+^ B6 background. CD69-deficient CD4 T cells were labelled with green fluorescent CFSE. These cells were mixed in the approximate ratio 1∶1 and injected i.v. into CD45.1^+^ B6 mice. 18 or 72 h later cells were isolated from blood, mesenteric lymph nodes (MLN), small intestinal lamina propria (siLP) and colonic lamina propria (cLP) of the hosts and analyzed for the surface expression of CD4, CD45.2, DsRed and CFSE by multicolour flow cytometry (FCM). Numbers of DsRed and CFSE expressing CD45.2^+^ CD4^+^ cells recovered 18 h (**A**) or 72 h (**D**) after the transfer from the host tissues were determined with FCM and presented as mean (± SEM) total number of recovered cells per tissue for four mice analyzed. N.S. – not statistically significant; *p≤0.05. Homing index (HI) for every tissue 18 h (**B**) or 72 h (**E**) post-injection is calculated as: HI = number of CD4^+^CD45.2^+^CFSE^+^ cells/number of CD4^+^CD45.2^+^DsRed^+^ cells : IR (where IR is input ratio calculated before the injection as: IR = number of CD4^+^CD45.2^+^CFSE^+^ cells/number of CD4^+^CD45.2^+^DsRed^+^ cells). This value was normalized to the HI in the blood, so the potential retention of the injected cells in some of the periphery organs is eliminated. Mean (± SEM) of blood-normalized HI per tissue for four mice is presented. The deviation from the theoretical mean (TM = 1) was assessed (*p≤0.05). **C.** 18 h after the cell transfer sections of small intestine and colon of the host were stained with Alexa Fluor 350-conjugated wheat germ agglutinin (blue) analyzed for the number of green (CFSE^+^ CD69^−/−^) and red (DsRed^+^ B6) CD4 T cells by confocal microscope (original magnification×40).

To compare the migration pattern of B6 and CD69^−/−^ CD4 T cells, we performed an *in vivo* competitive homing assay. Equal numbers of red DsRed^+^ B6 and green CFSE^+^ CD69^−/−^ CD4 T cells were transferred into the B6 mice. For easier detection of transferred lymphocytes the donor cells were expressing the common leukocyte antigen isotype CD45.2 and the host cells the isotype CD45.1. Lymphocytes were isolated 18 or 72 h post-transfer from the blood and intestinal tissues (MLN, siLP and cLP) of the hosts and the numbers of transferred DsRed^+^ B6 or CFSE^+^ CD69^−/−^ cells among the CD4^+^ CD45.2^+^ cell population were determined by FCM. 18 h after transfer the number of B6 and CD69-deficient CD4 T cells in the blood was the same while in MLN, siLP and cLP the number of CD69^−/−^ CD4 T cells was significantly increased (**Fig. 3A**). These results are confirmed for small intestinal and colonic tissue by *ex vivo* confocal imaging as we observed higher number of green CD69^−/−^ than red B6 CD4 T cells in both tissue samples (**Fig. 3C**). 72 h after the cell transfer significantly increased numbers of CD69-deficient cells were found in the intestinal tissues but also in blood (**Fig. 3D**). Possible retention of B6 CD4 T cells in some of the organs non-analysed here could contribute to the observed results. To eliminate this effect of tissue cell retention, we expressed the results of this assay also as homing index (HI). HI for intestinal tissues were calculated as the ratio of CD69^−/−^ to the B6 CD4 T cell number recovered from the hosts normalized to the IR of the injected cells and also normalized to the blood HI. This analyzes showed that 18 h post-transfer CD69^−/−^ CD4 T cells have an increased potential to migrate to the siLP and cLP, but not to MLN (**Fig. 3B**). 72 h after the cell transfer CD69-deficient CD4 T cells had a reduced potential to migrate to the MLN and same potential to migrate to siLP and cLP when compared to B6 CD4 T cells (**Fig. 3E**). This assay showed that CD69-deficient CD4 T cells migrate more efficiently than B6 cells to the intestinal tissues at early, but not late time point.

Cells on OT-II background showed slightly different migratory behaviour in this assay. 72 h after the DsRed OT-II/CFSE^+^ OT-II×CD69^−/−^ CD4 T cells were transferred into hosts, no difference in cell number could be observed in any tissue analysed (**Fig. S3**). When transferred mice were fed the specific antigen, OVA, increased number of CD69-deficeint OT-II CD4 T cells was found in siLP (**Fig. S3**). This means that homing of OT-II CD4 T cells to intestinal tissues depends not only on CD69, but also on presence of the antigen.

### CD69 Regulates Naïve/memory Cell Ratio in CD4 T cell Population

**Figure 4:**
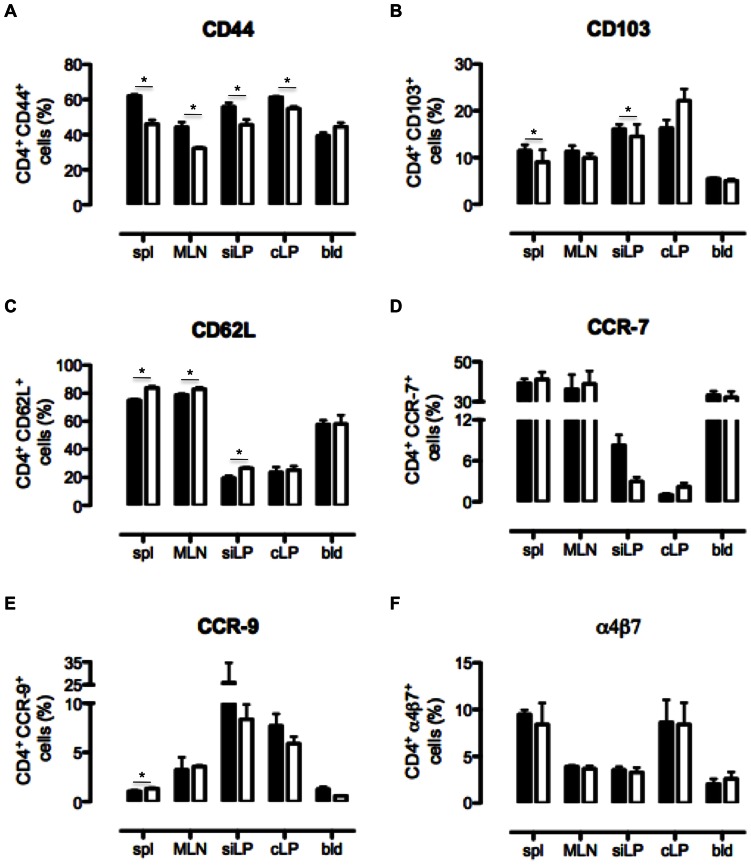
B6 and CD69^−/−^ CD4 T cells differ in the expression of naïve and memory surface cell markers. Cells were isolated from the spleen (spl), mesenteric lymph nodes (MLN), small intestinal lamina propria (siLP), colonic lamina propria (cLP) and blood (bld) of non-treated B6 and CD69^−/−^ mice and analyzed by flow cytometry. The surface expression of CD44 (**A**), CD103 (**B**), CD62L (**C**), CCR-7 (**D**), CCR-9 (**E**) and α4β7 integrin (**F**) by CD4 T cells were analysed. Graphs represent mean (± SEM) of CD4 T cell fraction expressing the indicated molecule for four mice per each strain per tissue. Black bars represent data for B6 and white bars for CD69^−/−^ cells. *p≤0.05.

Different migration patterns of B6 and CD69^−/−^ CD4 T cells observed *in vivo* could be the consequence of different expression of naïve and memory surface cell markers. Therefore, we investigated by FCM the expression of these markers on B6 and CD69^−/−^ CD4 T cells. Absence of CD69 significantly reduced the expression of memory cell markers in CD4 T cell population: CD44 in all tissues tested except of blood and CD103 in spleen and siLP (**Fig. 4A,B**). On the other hand, the expression of CD62L, marker of naïve cells and the chemotactic receptor that regulates the migration to lymph nodes and intestine, was increased in spleen, MLN and siLP of CD69^−/−^ mice (**Fig. 4C**). The expression of another naïve cell marker, CCR-7, did not significantly differ between B6 and CD69^−/−^ CD4 T cells (**Fig. 4D**). The expression of CCR-9, an intestine homing marker, was increased only in the spleen of CD69-deficient mice (**Fig. 4E**), while no differences were observed in the expression of α4β7 (**Fig. 4F**). These data indicated that the expression of main intestine homing markers is generally not affected by CD69. However, CD69 regulates naïve/memory cell ratio with CD69^−/−^ mice having a reduced fraction of memory CD44^+^ and CD103^+^ CD4 T cells and increased fraction of naïve CD62L^+^ CD4 T cells.

### Severe Colitis Induced in CD69^−/−^ Mice after the DSS Administration Depends on Increased Accumulation of CD4 T cells in cLP Attracted by CCL-1, CXCL-10 and CCL-19

**Figure 5:**
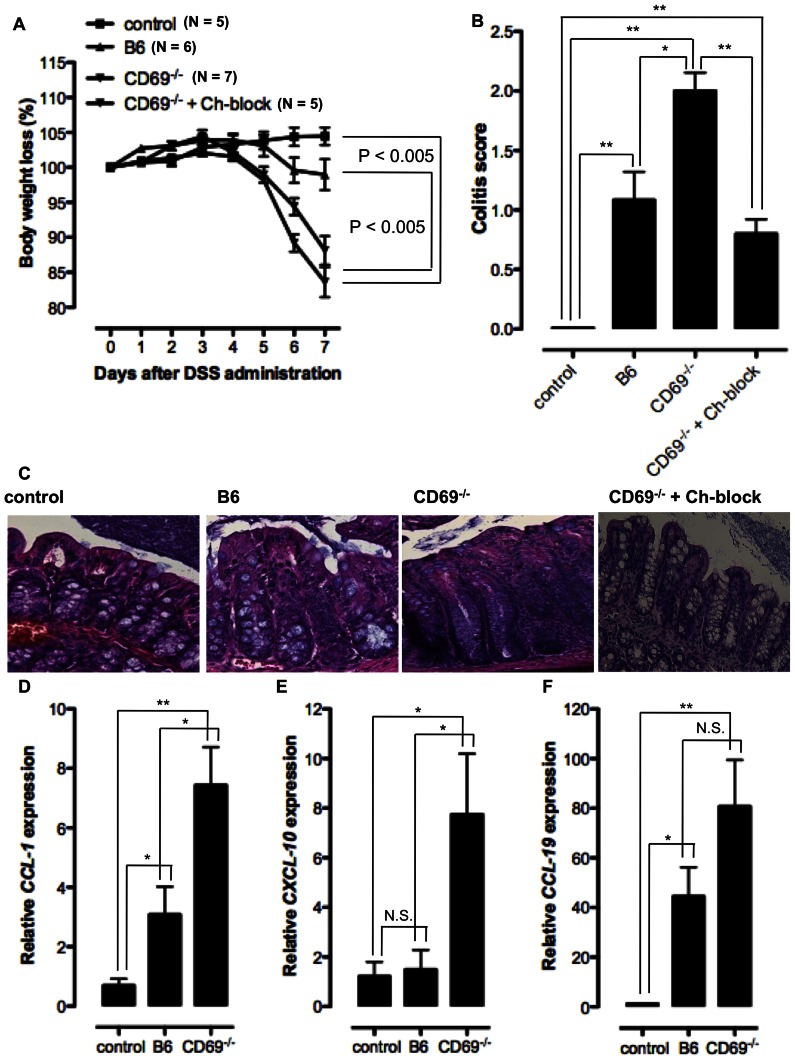
Increased susceptibility of CD69^−/−^ mice to DSS induced colitis is partially due to increased chemokine-dependent CD4 T cell migration to cLP. B6 or CD69^−/−^ mice are administrated 2% dextrane sodium sulphate (DSS) in the drinking water for 5 days and then provided with normal sterile water. One group of DSS-administrated CD69^−/−^ mice were treated with the mixture of CCL-1, CXCL-10 and CCL-19 blocking Abs by i.p. injection at days 0, 2, 4 and 6 (CD69^−/−^+Ch-block group). The control group represents non-treated B6 animals. **A.** Mean (± SEM) of body weight loss (%) of at least 5 mice per group is shown. **B.** Colitis scores of the histological colon sections of DSS treated B6 and CD69^−/−^ mice, control and CD69^−/−^+Ch-block groups. Mean (± SEM) for at least five mice per group are presented. **C.** Histopathological colon tissue samples taken from control B6 mice, DSS treated B6 or CD69^−/−^ animals and CD69^−/−^+Ch-block group were embedded in paraffin, sectioned on a microtome, mounted on slides, and stained with H&E. Representative images of one individual mouse per group (from at least five mice per group analyzed) are shown (original magnification×20). RNA was isolated from the frozen colon tissue samples of control B6 animals and DSS treated B6 or CD69^−/−^ mice and reverse transcribed to complementary DNA. Relative expression of *CCL-1* (**D**), *CXCL-10* (**E**) and *CCL-19* (**F**) genes compared to *β-actin* gene is measured by qRT-PCR. Mean (± SEM) for at least five mice per group are presented. N.S. – not statistically significant; *p≤0.05; **p≤0.005.

Administration of 2% DSS in the drinking water induced severe colitis in CD69-deficient mice as observed by the accelerated body weight loss compared to the DSS treated B6 animals and non-treated control group (**Fig. 5A**). Severity of intestinal inflammation is evaluated by histopathological examination of the colon. Average colitis score was significantly increased in the group of DSS-treated CD69^−/−^ mice when compared to the DSS-treated B6 and non-treated control groups (**Fig. 5B**). Colonic tissue of the control mice showed normal architecture and cellularity on the histological slides, while after the DSS treatment in B6 animals the signs of mild inflammation were observed (**Fig. 5C**). In CD69^−/−^ mice a massive infiltration of inflammatory cells and the los of tissue architecture confirmed the severity of the disease (**Fig. 5C**). Relative quantification of gene expression by RT-PCR was conducted using the RNA isolated from the frozen tissue sections of proximal colon taken from DSS administrated B6 and CD69^−/−^ mice and non-treated control animals. These analyzes revealed significant increase in the expression of *CCL-1*, *CXCL-10* and *CCL-19* in the inflamed colon tissue of DSS-treated CD69^−/−^ mice when compared to the other experimental groups (**Fig. 5D–F**). The treatment of DSS-induced colitis in CD69^−/−^ mice with the mixture of Abs that neutralize CCL-1, CXCL-10 and CCL-19 did not affect the accelerated body weight loss (**Fig. 5A**), but it reduced significantly the histopathological severity of the disease (**Fig. 5B**). CD69-deficient animals treated with neutralizing Abs showed the signs of mild colitis, with normal number of goblet cells, slightly elongated colonic crypts and few infiltrating inflammatory cells (**Fig. 5C**). Severe colonic inflammation with infiltration of high number of inflammatory cells into the colonic wall observed in CD69^−/−^ mice after DSS administration could be caused by the increased expression of pro-inflammatory chemokine ligands.

### High pro-inflammatory Cytokine Response and Early Influx of CD4 T cells Devoid of Foxp3 Treg Cells into cLP are Observed in DSS-treated CD69^−/−^ Mice

**Figure 6:**
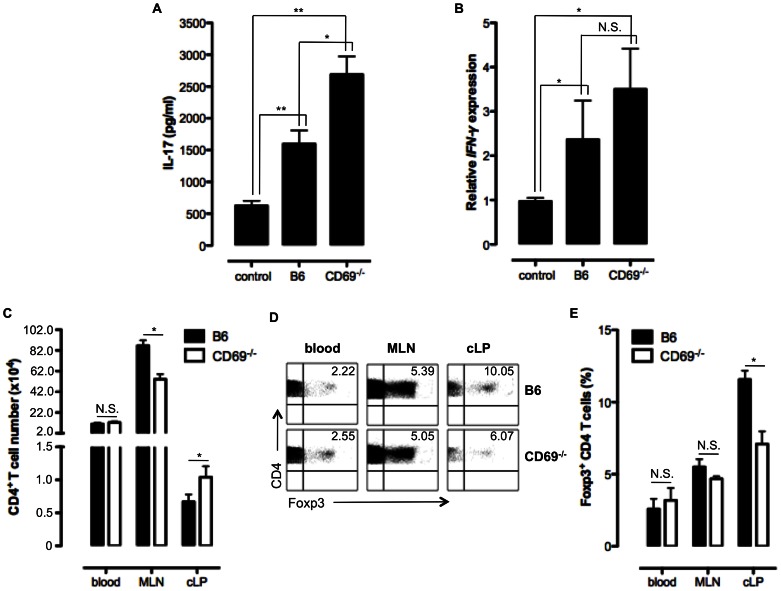
Severe colitis in DSS treated CD69^−/−^ mice is associated with increased pro-inflammatory response and early influx of CD4 T cells devoid of Foxp3 Treg cell into the colon. **A.** IL-17 concentration was determined by ELISA in the sera of control B6 mice and DSS treated B6 and CD69^−/−^ mice 7 days after the DSS administration. Mean (± SEM) of at least five mice per group is presented. **B.** RNA was isolated from frozen colon tissue samples of B6 and CD69^−/−^ mice treated with DSS, seven days after the DSS administration, or the control B6 mice and reverse transcribed to cDNA. Relative expression of *IFN-γ* gene was measured by qRT-PCR and presented as mean ± SEM of at least five mice per group. **C.** Mean (± SEM) total number of CD4^+^ T cells in blood, mesenteric lymph nodes (MLN) and colonic lamina propria (cLP) of 3 days DSS treated B6 and CD69^−/−^ mice are shown. At least five mice per each strain were analysed. **D.** The expression of Foxp3 by CD4 T cells from blood, MLN and cLP of B6 and CD69^−/−^ mice treated with DSS for 3 days was analysed by flow cytometry. Data from an individual, representative mouse (out of at least 5 mice per group analysed) are shown. Numbers indicate the percentage of CD4 T cells that express the transcriptional factor Foxp3. **E.** Mean (± SEM) total number of CD4^+^ Foxp3^+^ T cells in blood, MLN and cLP of 3 days DSS treated B6 and CD69^−/−^ mice are shown. At least five mice per each strain were analyzed. N.S. – not statistically significant; *p≤0.05; **p≤0.005.

To investigate the influence of the factors other than chemokines in DSS colitis model, we analyzed expression/production of pro-inflammatory cytokines as well. We saw that DSS-administrated CD69-deficient mice have significantly increased serum level of IL-17 as compared to the DSS-treated B6 and non-treated control group (**Fig. 6A**). Also, expression of the gene encoding pro-inflammatory cytokine IFN-γ was increased in the colonic tissue of the CD69^−/−^ animals treated with DSS (**Fig. 6B**). Furthermore, three days after the DSS administration CD69^−/−^ mice had reduced number of CD4 T cells in MLN and increased number of these cells in cLP when compared to B6 animals (**Fig. 6C**). Further analyses of these CD4 T cells showed that 3 days after the DSS administration CD4 T cell population in cLP of CD69-deficient mice contained significantly reduced fraction of Foxp3-expressing Treg cells compared to B6 mice, while in blood and MLN there was no difference (**Fig. 6D,E**). This early influx of CD4 T cells devoid of Treg cells together with the increased levels of pro-inflammatory cytokines contribute to the severe pathology observed in the colon of CD69^−/−^ mice treated with DSS.

### OT-II×CD69^−/−^ CD4 T cells Accumulate in cLP During Transfer Colitis Model

**Figure 7:**
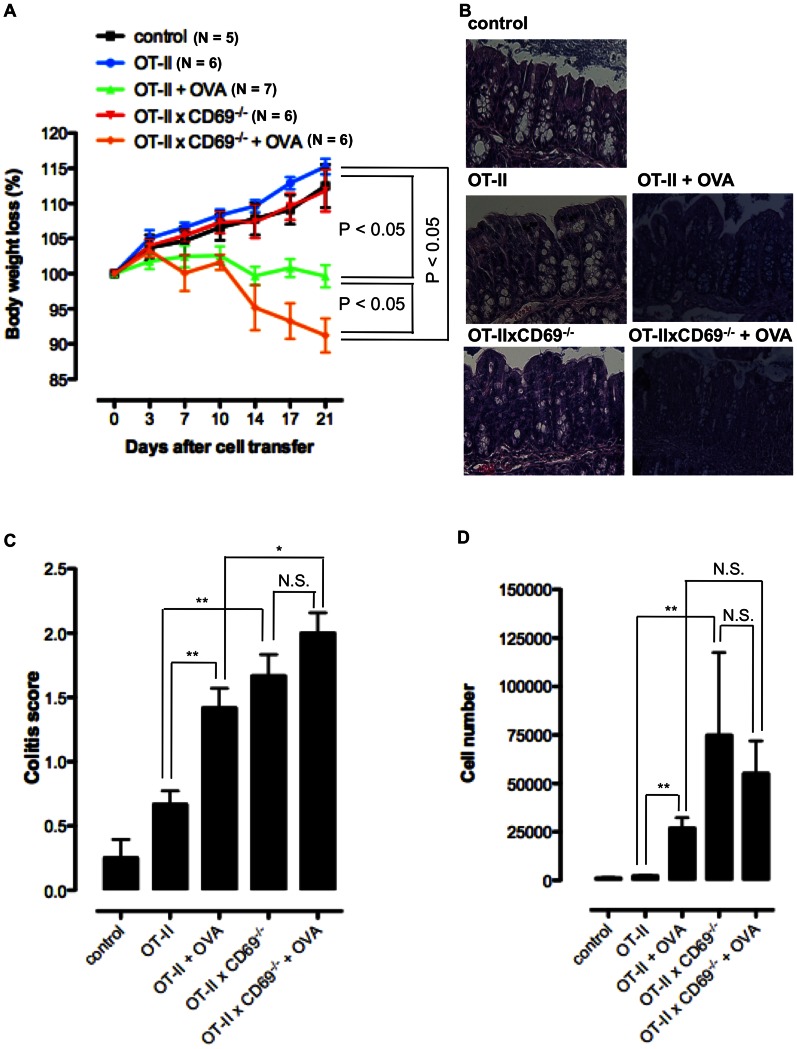
CD69-defficient OT-II CD4 T cells accumulate in cLP of RAG^−/−^ hosts during an antigen specific transfer colitis. Antigen specific colitis was induced by the transfer of CD45RB^high^ CD4 T cells, isolated from the spleen of OT-II or OT-II×CD69^−/−^ mice, into the RAG^−/−^ animals. Cell transfer was in some groups followed by the intragastrical feeding of the hosts with 1 mg OVA protein every second day. **A.** Mean (± SEM) of body weight loss (%) of at least five mice per group is shown for non-treated RAG^−/−^ animals (control group), hosts transferred with OT-II or OT-II×CD69^−/−^ cells and not fed with OVA (OT-II and OT-II×CD69^−/−^ groups) and host transferred with OT-II or OT-II×CD69^−/−^ cells and fed with OVA (OT-II+OVA and OT-II×CD69^−/−^+OVA groups). **B.** Histopathological colon tissue samples taken from the control, OT-II, OT-II×CD69^−/−^, OT-II+OVA and OT-II×CD69^−/−^+OVA groups were embedded in paraffin, sectioned on a microtome, mounted on slides, and stained with H&E. Representative images of one individual mouse from at least five mice per group analysed are shown (original magnification×20). **C.** Colitis scores of the histological colon sections of control animals and cell transferred hosts. Mean (± SEM) for at least five mice per group are presented. **D.** Cells recovered from the colonic lamina propria of the control mice and cell transferred hosts were analysed for the number of CD4^+^ T cells by flow cytometry. Mean (± SEM) for at least five mice per group are presented. *p≤0.05; **p≤0.005; N.S. – not significant.

Antigen-specific transfer colitis model was established to study the inflammatory response of CD4 T cells in the presence or absence of specific antigen in intestinal lumen. CD45RB^high^ CD4 T cells were sorted from the splenocytes of OT-II or OT-II×CD69^−/−^ mice and adoptively transferred into the RAG^−/−^ hosts. After the transfer hosts were fed or not every second day with 1 mg OVA protein. Both groups fed with OVA developed colitis, but the disease was more severe in the hosts transferred with OT-II×CD69^−/−^ cells and fed with OVA as showed by accelerated body weight loss compared to all the other groups (**Fig. 7A**) and by severe histopathology in colon (**Fig. 7B,C**). The hosts transferred with OT-II or OT-II×CD69^−/−^ CD45RB^high^ CD4 T cells that were not fed with OVA, normally gain body weight as the control group (**Fig. 7A**), although OT-II×CD69^−/−^ cell-transferred mice showed more severe histopathology in colonic tissue (**Fig. 7B,C**). Total number of OT-II CD4 T cells in cLP of the hosts was significantly increased compared to the control in the presence of antigen (**Fig. 7D**). The number of cLP OT-II×CD69^−/−^ CD4 T cells was increased significantly in both groups fed or not fed with OVA when compared to the control group (**Fig. 7D**). These data indicated that the accumulation of OT-II CD4 T cells in cLP is CD69-dependent in antigen-specific transfer model of colitis.

## Discussion

The data presented in this study indicated that the activation marker CD69 is involved in the regulation of chemokine expression. CD69-deficient mice showed increased transcript levels of some chemokine genes, such as *CCL-1*, *CXCL-10* and *CCL-19* in steady-state conditions and after colitis induction. We saw that CD69 regulates the expression of chemokine genes in both CD4^+^ and CD4^−^ cells. The portion of chemokines secreted by CD4^+^ T cells is considered to be minor, but it can still be important for the functions of immune cells [41], [42]. *In vitro* chemotactic assay showed that CD69^−/−^ CD4 T cells respond more efficiently to the chemokine stimuli compared to B6 CD4 T cells. This could be the result of increased chemokine receptor expression or, most probably, increased receptor affinity in CD69-deficient CD4 T cells. Hence, not only that CD69 regulates the chemokines expression, but it also modulates the ability of CD4 T cells to respond to stimulation with respective chemokine. However, the expression of some chemokines was not affected by CD69 and also CD69^−/−^ CD4 T cells responded the same as B6 CD4 T cells to the *in vitro* stimulation by CCL-4. The mechanisms for this selectivity in CD69-dependent chemokine responses are not known. Identification of intracellular pathways downstream of CD69 would help resolving this question.

In *in vivo* competitive homing assay, CD69-deficient CD4 T cells migrated more efficiently to the intestine at early time points after adoptive cell transfer into hosts. This could be explained by very efficient response to chemokine attraction or by increased expression of CCR-9 and CD62L by splenic CD69^−/−^ CD4 T cells used in this assay. CCR-9 is a well defined intestinal homing receptor [43], [44] while CD62L regulates lymphocyte homing to peripheral lymph nodes [45] and binds also to MadCAM-1 expressed in the intestine [46]; possible these molecules may be involved in the results we observed in *in vivo* competitive homing assays. This assay also showed that at later time points CD69^−/−^ cells have reduced homing index to MLN and that they are abound in the blood. This could be the result of their inability to down-modulate S1P_1_ receptor and stay in the lymphoid organs. Feeding the hosts with specific antigen after the cell transfer resulted in the increased migration of CD69^−/−^ CD4 T cells to the siLP, the place where antigen was located. Therefore, the presence of specific antigen is also important in CD4 T cell homing to the intestine. Possible differences in cell proliferation and retention, especially at later time points, and also possible effect of different fluorescent markers (DsRed and CFSE) on cell migration cannot be excluded in this assay.

Recently CD69 has been recognized as a marker of tissue resident memory T cell population [47], [48], [49], [50]. In this study we showed for the first time that CD69^−/−^ mice have decreased fraction of CD44^+^ and CD103^+^ memory CD4 T cells in peripheral tissues. These data confirmed that CD69 is important for the development of peripheral memory cell pool. Furthermore, disturbed naïve/memory cell ratios can change the migratory pattern of CD4 T cells and can be one of the factors contributing to the effects observed in this study.

Using two different colitis models we showed that CD69^−/−^ CD4 T cells accumulate preferably in the cLP under the inflammatory conditions. In DSS colitis model this was at least partially dependent on the increased expression levels of the chemokines *CCL-1*, *CXCL-10* and *CCL-19* in the colon of CD69-deficient animals, as neutralization of these three chemokines significantly improved the histopathological picture in colon. However, the chemokine-blocking treatment did not affect the body weight loss in DSS colitis model, indicating that other factors such as increased production of pro-inflammatory cytokines and decreased cLP Foxp3 Treg cell fraction play an important role in the disease development. In antigen specific colitis model OT-II×CD69^−/−^ CD4 T cells accumulated in cLP with or without antigen presence showing that this effect is only CD69-dependend. However, it is not clear weather this accumulation is a result of cell migration or proliferation, as the effect of CD69 on cell proliferation is not known.

Our study provides first evidence that CD69 does not regulate the lymphocyte migration only by influencing S1P_1_ expression, but also by regulating the expression of chemokines and naïve/memory T cell markers. We also showed that absence of CD69 leads to the accumulation of CD4 T cells in the intestinal mucosa. We hypothesize that in physiological conditions the up-regulation of CD69 on activated CD4 T cells in the intestine acts as a negative feedback mechanism to prevent the excessive inflammation by reducing the amounts of produced pro-inflammatory chemokines and cytokines and by increasing the fraction of Foxp3 Treg cells and memory cells. Our study indicated that CD69 regulates the immune system in very complex ways and showed the need for further studies that will determine the mechanisms by which CD69 regulates the functions of lymphocytes.

## Supporting Information

Figure S1 **Absence of CD69 does not affect the expression of** ***CCL-5*** **and** ***CCL-8*** **.** RNA was isolated from the frozen mesenteric lymph nodes (MLN), small intestinal (si) and colonic (co) tissue samples or from sorted CD4^+^ and CD4^−^ spleen cells of non-treated B6 or CD69^−/−^ animals and reverse transcribed to complementary DNA. Relative expression of *CCL-5* (**A** and **C**) and *CCL-8* (**B** and **D**) as compared to *β-actin* gene is analysed by qRT-PCR. Mean (± SEM) for at least six mice per each strain is presented. N.S. – not significant
(TIF)

Figure S2 **B6 and CD69^−/−^ mice do not differ in the surface expression of CXCR-3 receptor by CD4 T cells.** Cells were isolated from the spleen (spl), mesenteric lymph nodes (MLN), small intestinal lamina propria (siLP), colonic lamina propria (cLP) and blood (bld) of non-treated B6 and CD69^−/−^ mice and analyzed by flow cytometry. Graph represents mean (± SEM) of CD4 T cell fraction expressing CXCR-3 for four mice per each strain per tissue. *p≤0.05
(TIF)

Figure S3 **OT-II×CD69^−/−^ CD4 T cells are homing in the higher numbers to the small intestine in the presence of antigen.** CD4 T cells were enriched from the spleen of transgenic OT-II**×**DsRed or OT-II**×**CD69^−/−^ mice (both on Vβ5^+^ background). CD69-deficient cells were labelled with CSFE. Red fluorescent OT-II×DsRed and green fluorescent CFSE^+^ OT-II×CD69^−/−^ CD4 T cells were mixed in the ratio 1∶1 and transferred to the B6 hosts. Host were fed or not intragastrically with 1 mg ovalbumin protein daily. Three days after, the cells were obtained from the blood, mesenteric lymph nodes (MLN), small intestinal lamina propria (siLP) and colonic lamina propria (cLP) of the hosts. **A.** The number of DsRed^+^ OT-II and CFSE^+^ OT-II×CD69^−/−^ cells among CD4^+^Vβ5^+^ cell population was determined in the tissues of the hosts by flow cytometry and presented as mean (± SEM) total number of recovered cells per tissue for five mice analyzed. N.S. – not statistically significant; *p≤0.05. **B.** Homing index (HI) for every tissue is calculated as: HI = number of CD4^+^ Vβ5^+^CFSE^+^ cells/number of CD4^+^ Vβ5^+^DsRed^+^ cells: IR (where IR is input ratio calculated before the injection as: IR = number of CD4^+^ Vβ5^+^CFSE^+^ cells/number of CD4^+^ Vβ5^+^DsRed^+^ cells). HI for intestinal tissues was normalized to the HI in the blood to eliminate the potential retention of the injected cells in some of the periphery organs. Mean (± SEM) of blood-normalized HI per tissue for five mice is presented. The deviation from the theoretical mean (TM = 1) is assessed (*p≤0.05).
(TIF)

Table S1 **Expression of selected chemokine-related genes differentially expressed in CD69-activated compared to B6 CD4 T cells analyzed by microarray.**
(DOCX)

Table S2 **Expression of selected chemokine-related genes differentially expressed in CD69^−/−^ compared to B6 CD4 T cells analyzed by microarray.**
(DOCX)
