# Supplementary figures and images for: The Early Activation Marker CD69 Regulates the Expression of Chemokines and CD4 T Cell Accumulation in Intestine
Source: PLoS One. 2013 Jun 12;8(6):e65413. doi: 10.1371/journal.pone.0065413 (PMC3680485; doi:10.1371/journal.pone.0065413)

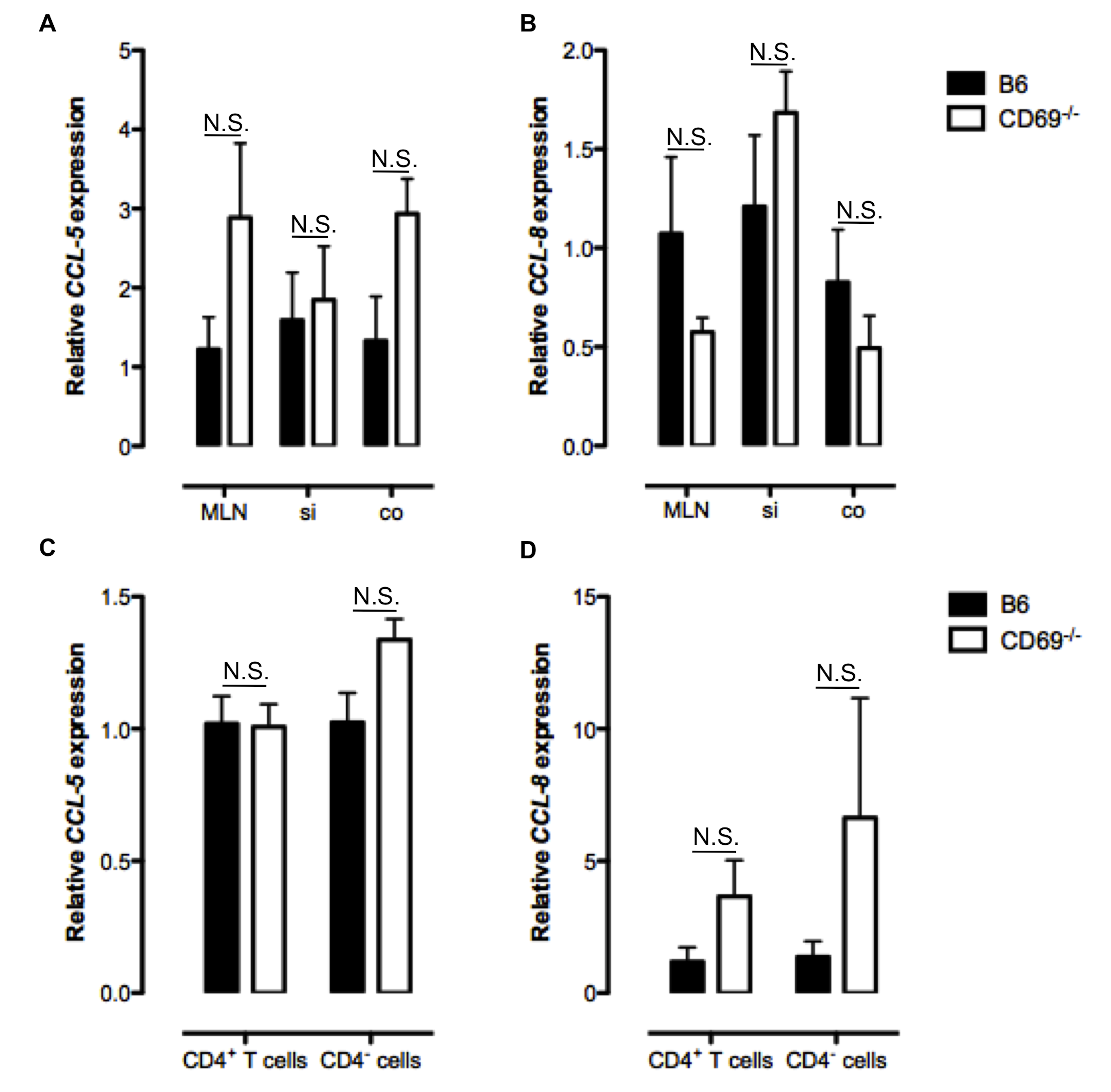

Supplement: Figure S1 — Absence of CD69 does not affect the expression of CCL-5 and CCL-8 . RNA was isolated from the frozen mesenteric lymph nodes (MLN), small intestinal (si) and colonic (co) tissue samples or from sorted CD4+ and CD4− spleen cells of non-treated B6 or CD69−/− animals and reverse transcribed to complementary DNA. Relative expression of CCL-5 (A and C) and CCL-8 (B and D) as compared to β-actin gene is analysed by qRT-PCR. Mean (± SEM) for at least six mice per each strain is presented. N.S. – not significant (TIF) [file pone.0065413.s001.tif]

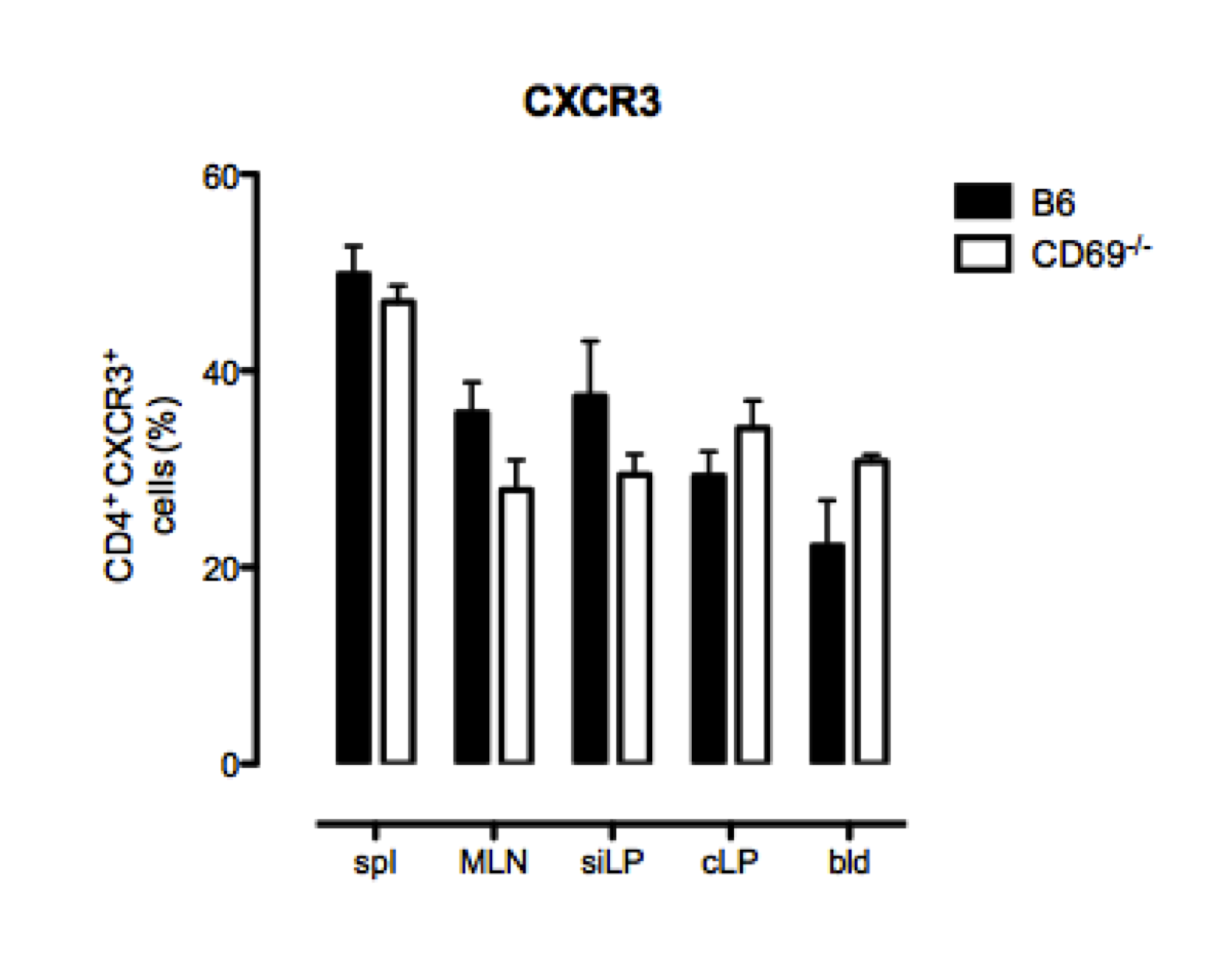

Supplement: Figure S2 — B6 and CD69−/− mice do not differ in the surface expression of CXCR-3 receptor by CD4 T cells. Cells were isolated from the spleen (spl), mesenteric lymph nodes (MLN), small intestinal lamina propria (siLP), colonic lamina propria (cLP) and blood (bld) of non-treated B6 and CD69−/− mice and analyzed by flow cytometry. Graph represents mean (± SEM) of CD4 T cell fraction expressing CXCR-3 for four mice per each strain per tissue. *p≤0.05 (TIF) [file pone.0065413.s002.tif]

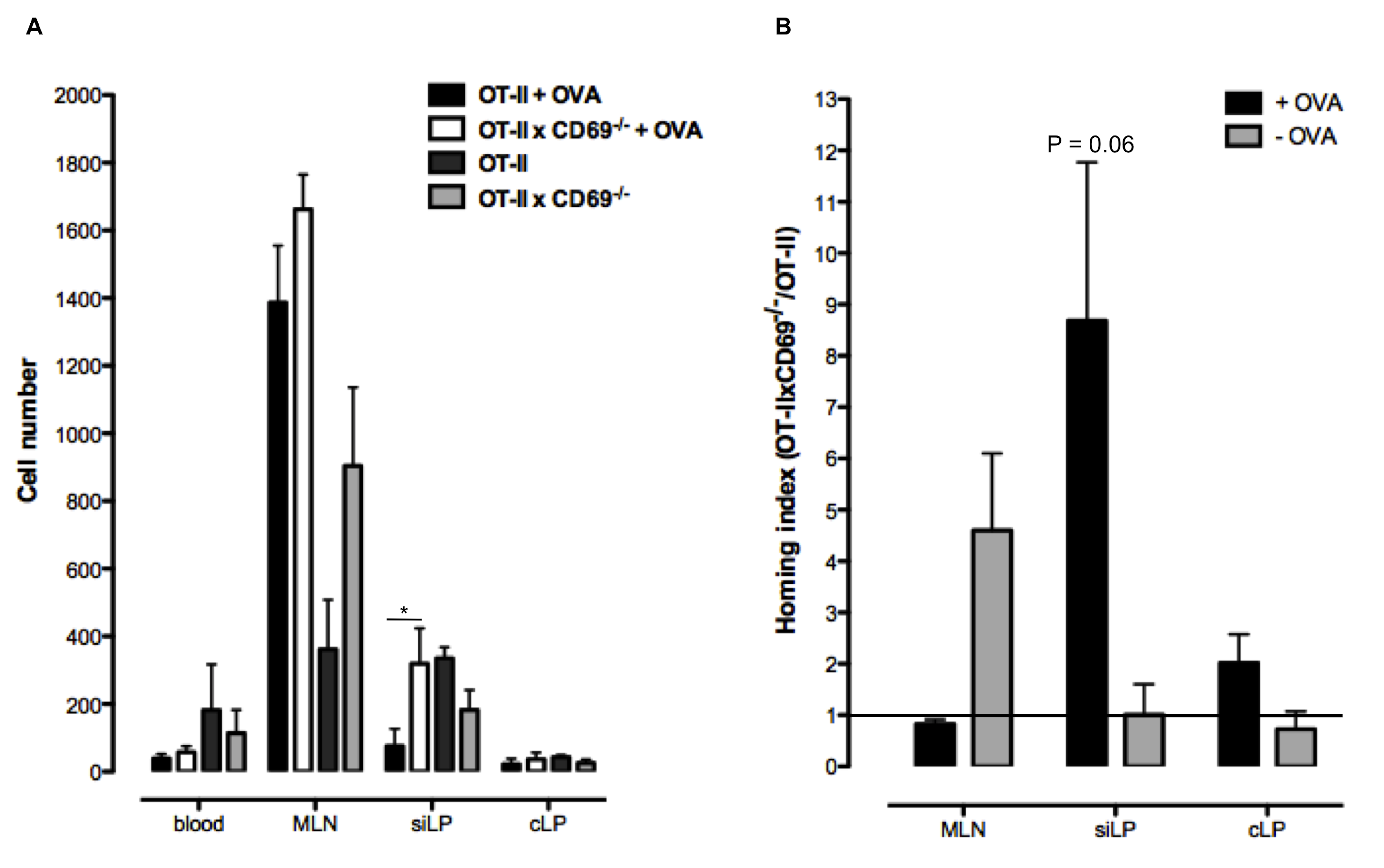

Supplement: Figure S3 — OT-II×CD69−/− CD4 T cells are homing in the higher numbers to the small intestine in the presence of antigen. CD4 T cells were enriched from the spleen of transgenic OT-II×DsRed or OT-II×CD69−/− mice (both on Vβ5+ background). CD69-deficient cells were labelled with CSFE. Red fluorescent OT-II×DsRed and green fluorescent CFSE+ OT-II×CD69−/− CD4 T cells were mixed in the ratio 1∶1 and transferred to the B6 hosts. Host were fed or not intragastrically with 1 mg ovalbumin protein daily. Three days after, the cells were obtained from the blood, mesenteric lymph nodes (MLN), small intestinal lamina propria (siLP) and colonic lamina propria (cLP) of the hosts. A. The number of DsRed+ OT-II and CFSE+ OT-II×CD69−/− cells among CD4+Vβ5+ cell population was determined in the tissues of the hosts by flow cytometry and presented as mean (± SEM) total number of recovered cells per tissue for five mice analyzed. N.S. – not statistically significant; *p≤0.05. B. Homing index (HI) for every tissue is calculated as: HI = number of CD4+ Vβ5+CFSE+ cells/number of CD4+ Vβ5+DsRed+ cells: IR (where IR is input ratio calculated before the injection as: IR = number of CD4+ Vβ5+CFSE+ cells/number of CD4+ Vβ5+DsRed+ cells). HI for intestinal tissues was normalized to the HI in the blood to eliminate the potential retention of the injected cells in some of the periphery organs. Mean (± SEM) of blood-normalized HI per tissue for five mice is presented. The deviation from the theoretical mean (TM = 1) is assessed (*p≤0.05). (TIF) [file pone.0065413.s003.tif]
