# Supplementary material for: The Early Activation Marker CD69 Regulates the Expression of Chemokines and CD4 T Cell Accumulation in Intestine
Source: PLoS One. 2013 Jun 12;8(6):e65413. doi: 10.1371/journal.pone.0065413 (PMC3680485; doi:10.1371/journal.pone.0065413)
Supplement: Table S1 — Expression of selected chemokine-related genes differentially expressed in CD69-activated compared to B6 CD4 T cells analyzed by microarray. (DOCX) [file pone.0065413.s004.docx]

**Table S1: Expression of selected chemokine-related genes differentially expressed in CD69-activated compared to B6 CD4 T cells analyzed by microarray**.

| **Gene symbol** | **Description** | **Fold-change (log2)** | **FDR** |
| --- | --- | --- | --- |
| Xcl1 | chemokine (C motif) ligand 1 | -3.07 | 6.05e-14 |
| Cxcl9 | chemokine (C-X-C motif) ligand 9 | -1.90 | 6.05e-14 |
| Cxcl10 | chemokine (C-X-C motif) ligand 10 | -1.12 | 6.05e-14 |
| Ccl3 | chemokine (C-C motif) ligand 3 | -1.12 | 6.05e-14 |
| Ccl9 | chemokine (C-C motif) ligand 9 | -0.81 | 0.0043 |
| Ccr8 | chemokine (C-C motif) receptor 8 | -0.73 | 6.05e-14 |
| Ccl4 | chemokine (C-C motif) ligand 4 | -0.70 | 0.019 |
| Ccl1 | chemokine (C-C motif) ligand 1 | -0.68 | 5.29e-05 |
| Ccr4 | chemokine (C-C motif) receptor 4 | -0.67 | 2.53e-11 |

False discovery rate (FDR) ≤ 0.05 was considered statistically significant.
