# Supplementary material for: The Early Activation Marker CD69 Regulates the Expression of Chemokines and CD4 T Cell Accumulation in Intestine
Source: PLoS One. 2013 Jun 12;8(6):e65413. doi: 10.1371/journal.pone.0065413 (PMC3680485; doi:10.1371/journal.pone.0065413)
Supplement: Table S2 — Expression of selected chemokine-related genes differentially expressed in CD69−/− compared to B6 CD4 T cells analyzed by microarray. (DOCX) [file pone.0065413.s005.docx]

**Table S2: Expression of selected chemokine-related genes differentially expressed in CD69^-/-^ compared to B6 CD4 T cells analyzed by microarray.**

| **Gene symbol** | **Description** | **Fold-change (log2)** | **FDR** |
| --- | --- | --- | --- |
| Ccr5 | chemokine (C-C motif) receptor 5 | 0.90 | 3.16e-14 |
| Ccr8 | chemokine (C-C motif) receptor 8 | 0.76 | 3.16e-14 |
| Ccl4 | chemokine (C-C motif) ligand 4 | 0.76 | 3.16e-14 |
| Cxcl10 | chemokine (C-X-C motif) ligand 10 | 0.63 | 1.19e-13 |
| Ccr4 | chemokine (C-C motif) receptor 4 | 0.41 | 0.00015 |
| Ccl3 | chemokine (C-C motif) ligand 3 | 0.33 | 8.71e-07 |

False dscovery rate (FDR) ≤ 0.05 was considered statistically significant.
